# CC-type glutaredoxins mediate plant response and signaling under nitrate starvation in *Arabidopsis*

**DOI:** 10.1186/s12870-018-1512-1

**Published:** 2018-11-13

**Authors:** Ji-Yul Jung, Ji Hoon Ahn, Daniel P. Schachtman

**Affiliations:** 1 Department of Life Sciences, Korea University, Seoul, 02841 South Korea; 2 Department of Agronomy and Horticulture, Center for Biotechnology, University of Nebraska Lincoln, Lincoln, NE 68588 USA

**Keywords:** Glutaredoxin, ROXY, Nitrate, Starvation, ROS, Signaling

## Abstract

**Background:**

Nitrogen is an essential nutrient in plants. Despite the importance of nitrogen for plant growth and agricultural productivity, signal transduction pathways in response to nitrate starvation have not been fully elucidated in plants.

**Results:**

Gene expression analysis and ectopic expression were used to discover that many CC-type glutaredoxins (*ROXYs*) are differentially expressed in response to nitrate deprivation. A gain-of-function approach showed that *ROXYs* may play a role in nutrient sensing through the regulation of chlorophyll content, root hair growth, and transcription of nitrate-related genes such as *NRT2.1* under low or high nitrate conditions. Reactive oxygen species (ROS) were produced in plant roots under nitrate starvation and H_2_O_2_ treatment differentially regulated the expression of the *ROXYs,* suggesting the involvement of ROS in signaling pathways under nitrate deficiency.

**Conclusion:**

This work adds to what is known about nitrogen sensing and signaling through the findings that the ROXYs and ROS are likely to be involved in the nitrate deprivation signaling pathway.

**Electronic supplementary material:**

The online version of this article (10.1186/s12870-018-1512-1) contains supplementary material, which is available to authorized users.

## Hightlight

CC-type glutaredoxins play roles in plant response and signaling under nitrate starvation conditions in *Arabidopsis* possibly acting downstream of reactive oxygen species.

## Background

Plants require nutrients to complete their life cycle. Among them, nitrogen is required in greater abundance and is essential for plant growth. Plant roots preferentially take up nitrate and ammonium from the soil although they have the capacity to absorb organic N sources, such as amino acids, in soils that contain high concentrations of organic matter [1, 2]. In aerobic soil conditions, ammonium and amino acids are poorly available and nitrate is relatively abundant in the anionic form which is readily dissolved in soil water. Therefore, plants tend to use nitrate as the main source of inorganic nitrogen [3–5]. Since nitrogen availability in plants can be affected by various environmental conditions, including soil pH, soil type, precipitation, and temperature [4, 6], plants frequently experience nitrogen deficiency, which can greatly reduce the yield of plants. Therefore, studying plant responses to nitrogen deficiency is of significant agricultural importance.

Although certain aspects of how plants respond to nitrogen deprivation at physiological and transcriptional levels are known, the details of the signaling pathways in response to low or high nitrogen are not well characterized [7–12]. Nitrogen starvation in plants induces both physiological changes of root structures and transcriptional changes of nitrogen-related genes such as *AMT1* ammonium transporters and *NRT2* nitrate transporters including *NRT2.1*, a main contributor in nitrate uptake during nitrogen starvation [12, 13]. Many factors are reported to be involved in low nitrogen-induced plant responses. These include transcription factors (LATERAL ORGAN BOUNDARY DOMAIN (LBD) 37/38/39 and NIN-LIKE PROTEIN (NLP) 7), components of peptide signaling (C-TERMINALLY ENCODED PEPTIDE (CEP), CEP RECEPTOR1/2 (CEPR1/2), CLAVATA3/ESR-RELATED (CLE) 1/3/4/7, and CLAVATA1 (CLV1)), microRNAs (miR169), and plant hormones (cytokinin and auxin) [12, 14–20]. Calcium signaling may be a component in low nitrogen signaling as CALCINEURIN B-LIKE PROTEIN 7 (CBL7) is involved in the regulation of root growth under nitrate limitation in *Arabidopsis* [21]. Reactive oxygen species (ROS) are also implicated in the low nitrate signaling because H_2_O_2_ concentrations in plant roots increased in response to nitrogen starvation [22, 23]. It is known that ROS as a signaling molecule mediates signal transduction in plants under the deprivation of some nutrients such as potassium [24], phosphate [25], and boron [26]. And NADPH oxidases are mainly responsible for low nutrient-induced ROS production based on chemical inhibitor and genetic studies [22, 27, 28].

Glutaredoxins (GRXs) are small ubiquitous proteins that are involved in disulphide bridge or protein-glutathione reduction in plant cells [29–36]. There are 31 GRX genes in *Arabidopsis* that can be classified into three distinct subgroups based on the amino acid sequences at their active sites: the CPYC, CGFS, and CC-type GRX classes [32, 35]. Out of the 31 GRXs in *Arabidopsis*, 21 GRXs belong to the CC-type class, which is specific for land plants, while the other two GRX classes, CPYC and CGFS, are common to eukaryotes and prokaryotes [31, 37–39]. Although the roles of GRXs have been associated mainly with oxidative stress [40–52], there is growing evidence that GRXs, especially CC-type GRXs, also play important roles in cell signaling and development [30, 33, 34, 53–60]. For example, three CC-type GRXs, ROXY1, ROXY2, and ROXY4, which interact with TGA transcription factors in the nucleus, are required for flower development [36, 54, 55, 61–64] and another CC-type GRX gene, *ROXY19*, has been demonstrated to act as a repressor in the detoxification pathways [65] and pathogen responses through salicylic acid (SA)/jasmonic acid (JA) signaling [36, 66, 67]. ROXY19 was also shown to act as an adapter protein for the assembly of transcriptional repressor complexes on TGA-regulated target promoters [68]. Recently, it was shown that ROXY11–13 and ROXY15 are involved in nitrate-induced primary root growth inhibition [57, 69].

Previous microarray studies indicated that many *ROXYs* are differentially expressed under abiotic stresses, suggesting the involvement of *ROXYs* in abiotic stress [70]. However, not much is known about the gene expression of *ROXYs* under low nutrient stress and the functional consequences of the changes in their expression. In this study, we quantified changes in the gene expression of *ROXYs* under low nutrient conditions by removing nitrogen from media. We found that a number of *ROXYs* are differentially regulated in response to nitrate starvation. Based on the phenotypes of overexpression lines of two *ROXYs* (*ROXY9* and *ROXY15*), evidence is provided showing a role for CC-type GRXs in nitrate starvation signaling. ROS appears to be another component that regulates the differential expression of *ROXYs* under nitrate deprivation conditions. This study provides important new information that further elucidates additional novel components in the complex nitrate signaling pathway in plants.

## Results

### Nitrate starvation differentially regulates the expression of *ROXYs*

**Fig. 1:**
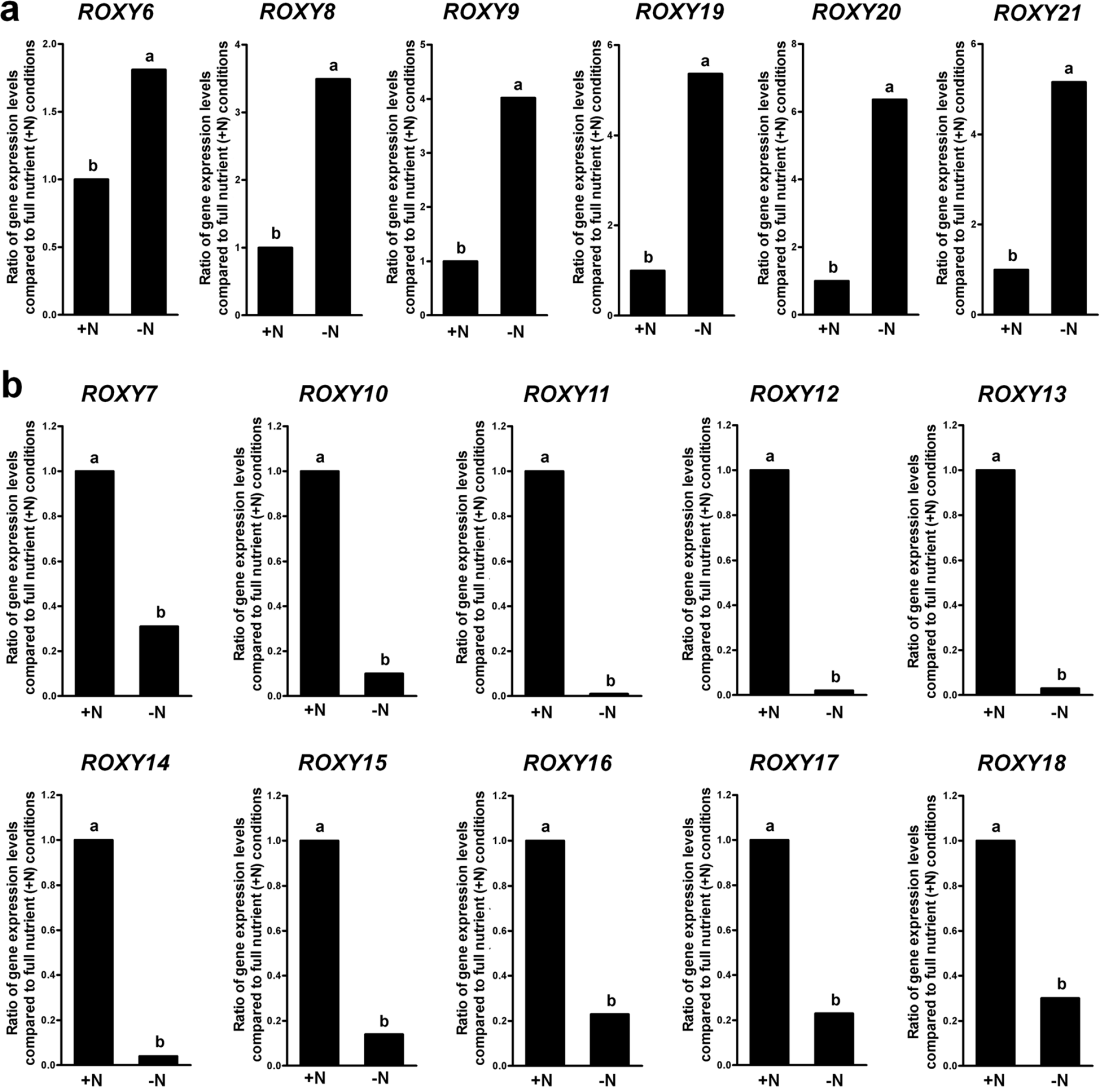
Expression levels of *ROXY* genes under nitrate-sufficient and nitrate-deficient conditions. **a** *ROXY6,8,9,19–21* are upregulated under nitrate-deficient conditions (-N). **b** *ROXY7,10–18* are downregulated under nitrate-deficient conditions. Seven-day-old seedlings grown under full nutrient conditions were treated with either 4 mM nitrate (+N) or no nitrate (-N) for 3 d. Expression levels of *ROXY* genes in the wild type Col-0 under no nitrate relative to those in the wild type seedlings under full nutrient conditions (set to the value of 1) are shown. The expression data were obtained by qRT-PCR. An *ACTIN7* was used as a reference gene. Different letters above the bars indicate values that are significantly different (*n* = 3 biological replicates, *P* < 0.05; *t* test)

In *Arabidopsis*, there are 21 CC-type *GRXs*, which have been named *ROXY1* to *ROXY21* [47]. To determine the expression pattern of *ROXYs* under various reduced nutrient conditions, quantitative real time PCR (qRT-PCR) was used to measure the *ROXYs* mRNA level in the wild type Columbia-0 seedlings grown on full nutrient medium (+N) and no nitrate medium (-N). As a nitrogen source in nutrient medium, we only used nitrate and not ammonium since ammonium is poorly available in actual soil conditions. Sixteen *ROXY* genes tested in this study showed altered transcriptional responses in plants under nitrate starvation. The expression of six *ROXYs* (*ROXY6,8,9,19–21*) was significantly upregulated by nitrate deficiency (Fig. 1a), while the expression of 10 *ROXYs* (*ROXY7,10–18*) was downregulated (Fig. 1b). These data suggest that *ROXY* genes may be involved in signaling pathways under nitrate deficiency conditions in plants.

### Possible roles of ROXYs under nitrate-sufficient or -deficient conditions

**Fig. 2:**
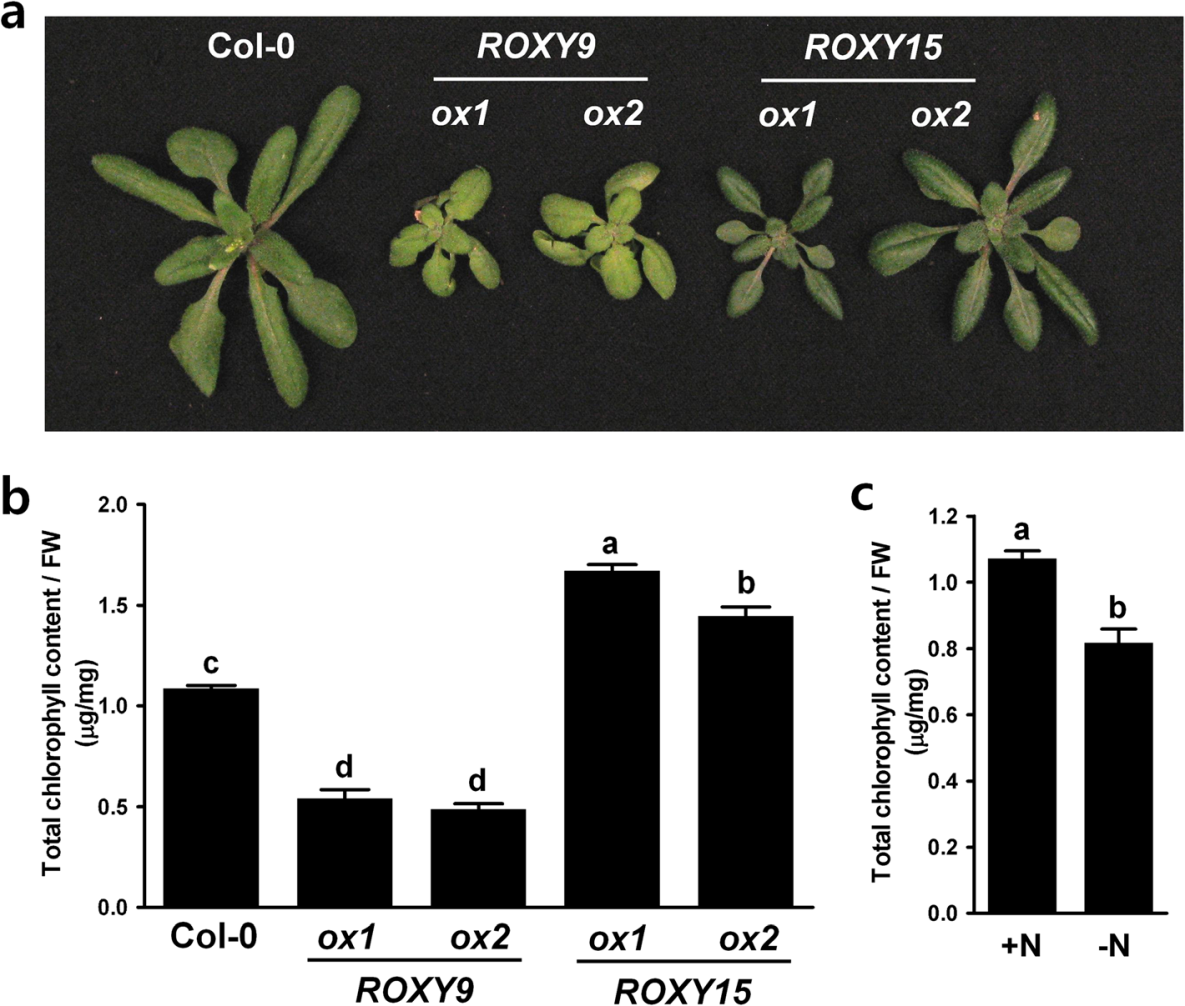
Overexpression of *ROXY9* reduces chlorophyll content while overexpression of *ROXY15* increases chlorophyll content. Images of shoots of three-week-old plants grown in the soil. **a** Wild-type plants and two independent transgenic plants overexpressing *ROXY9* or *ROXY15* (ox) were grown for three weeks in the soil and their leaf chlorophyll contents were measured as described in the Method section. **b** Total chlorophyll content of plants shown in (**a**). Fully developed mature leaves were used for the measurement of chlorophyll content. The total chlorophyll content was expressed as micrograms per milligram leaf fresh weight. Values are mean ± SE from two leaves per plant (*n* = 10 plants). Results from one of two independent experiments are shown. **c** Total chlorophyll content of wild type Col-0 plants deprived of nitrate. Ten-day-old seedlings grown on complete nutrient media were treated with either 4 mM nitrate (+N) or no nitrate (-N) for 4 days. Values are mean ± SE from 5 shoots per replicate (*n* = 5 replicates). Results from one of two independent experiments are shown. Different letters above the bars indicate values that are significantly different (*P* < 0.05; *t* test)

**Fig. 3:**
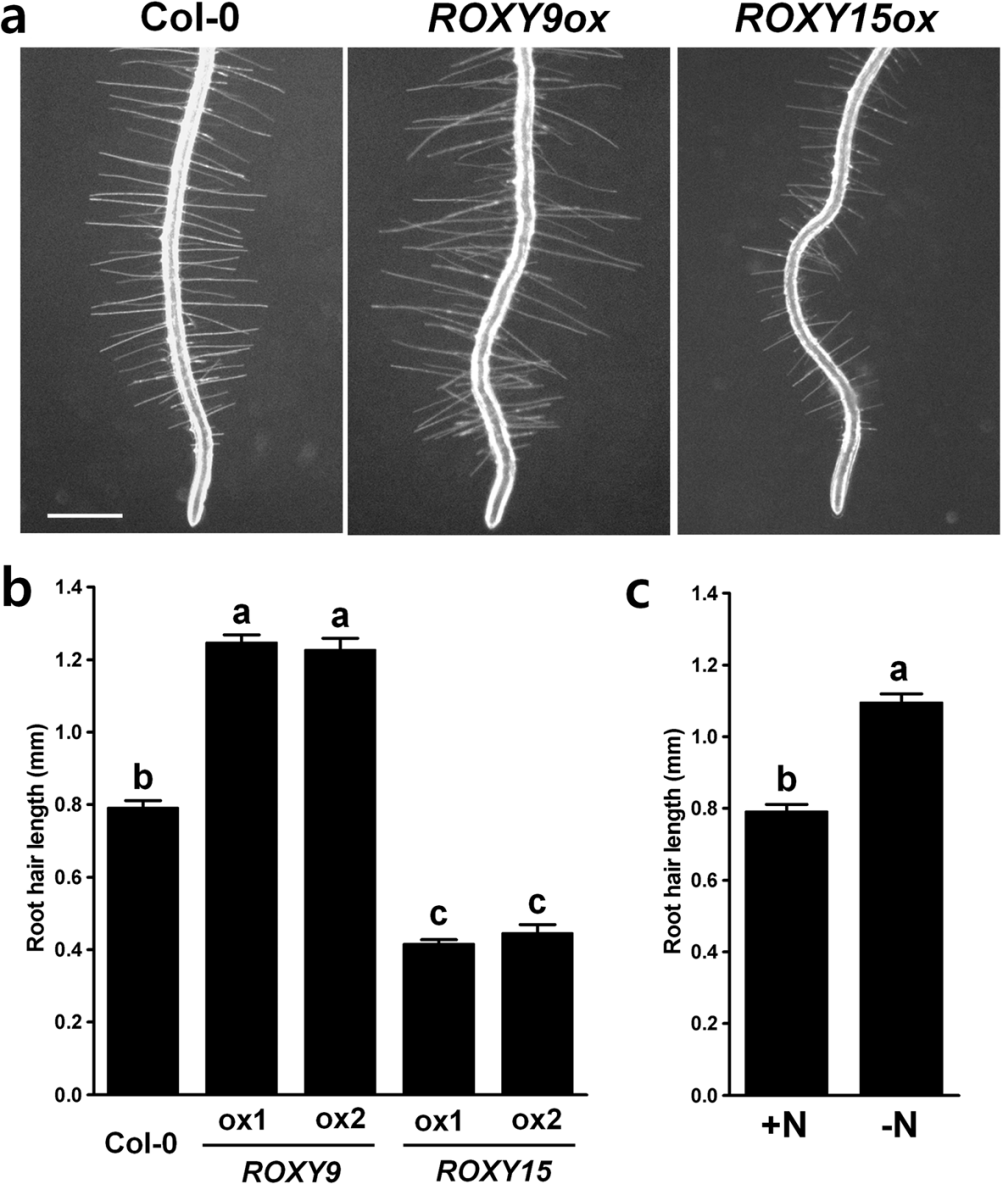
Overexpression of *ROXY9* stimulates root hair elongation while overexpression of *ROXY15* inhibits root hair elongation. (**a**, **b**) Light microscopy images showing root hairs of wild-type Col-0 seedlings and two independent transgenic seedlings overexpressing *ROXY9* or *ROXY15* (ox). Roots from four-day-old seedlings grown on nitrate-sufficient medium were used for the measurement of the root hair length. Bar = 0.5 mm. **b** Quantified data showing the root hair length of wild-type Col-0 seedlings and two independent transgenic seedlings overexpressing *ROXY9* or *ROXY15* (*n* = 10 seedlings, means ± SE). **c** Root hair length of wild type Col-0 seedlings grown under nitrate-sufficient (+N) and nitrate-deficient (-N) conditions (*n* = 10 seedlings, means ± SE). Four-day-old roots treated with either 4 mM nitrate or no nitrate for 24 h were used for the measurement of root hair length. Results from one of two independent experiments are shown here. Different letters above the bars indicate values that are significantly different (*P* < 0.05; *t* test)

Nitrate deprivation was shown to differentially regulate the expression of *ROXYs,* suggesting that these genes may play an important role under the nitrate starvation conditions. To further investigate the possible functional roles of ROXYs in response to nitrate deficiency, an overexpression approach was chosen due to the large size of the gene family and a potential issue related with redundancy making it difficult to reveal phenotypes of knockout lines. Two independent transgenic *Arabidopsis* lines overexpressing *ROXY9* or *ROXY15* (Additional file 1) were used for their phenotypic analyses. The reason we chose these two ROXY genes is that they showed the opposite transcriptional regulation under nitrate starvation: *ROXY9* is upregulated and *ROXY15* is downregulated by nitrate deprivation (Fig. 1). Compared with the wild type plants grown in soils, leaf chlorophyll content was significantly lower in the two independent lines overexpressing *ROXY9* and higher in the two independent lines overexpressing *ROXY15* (Fig. 2a, b). Wild type plants grown under nitrate starvation conditions had lower chlorophyll contents when compared with those grown under nitrate-sufficient conditions (Fig. 2c). Also compared with the wild type seedlings grown on full nutrient medium, the length of root hairs was significantly longer in the two independent lines overexpressing *ROXY9* and shorter in the two independent lines overexpressing *ROXY15* (Fig. 3a, b). And nitrate deficiency in wild type seedlings promoted root hair elongation (Fig. 3c).

**Fig. 4:**
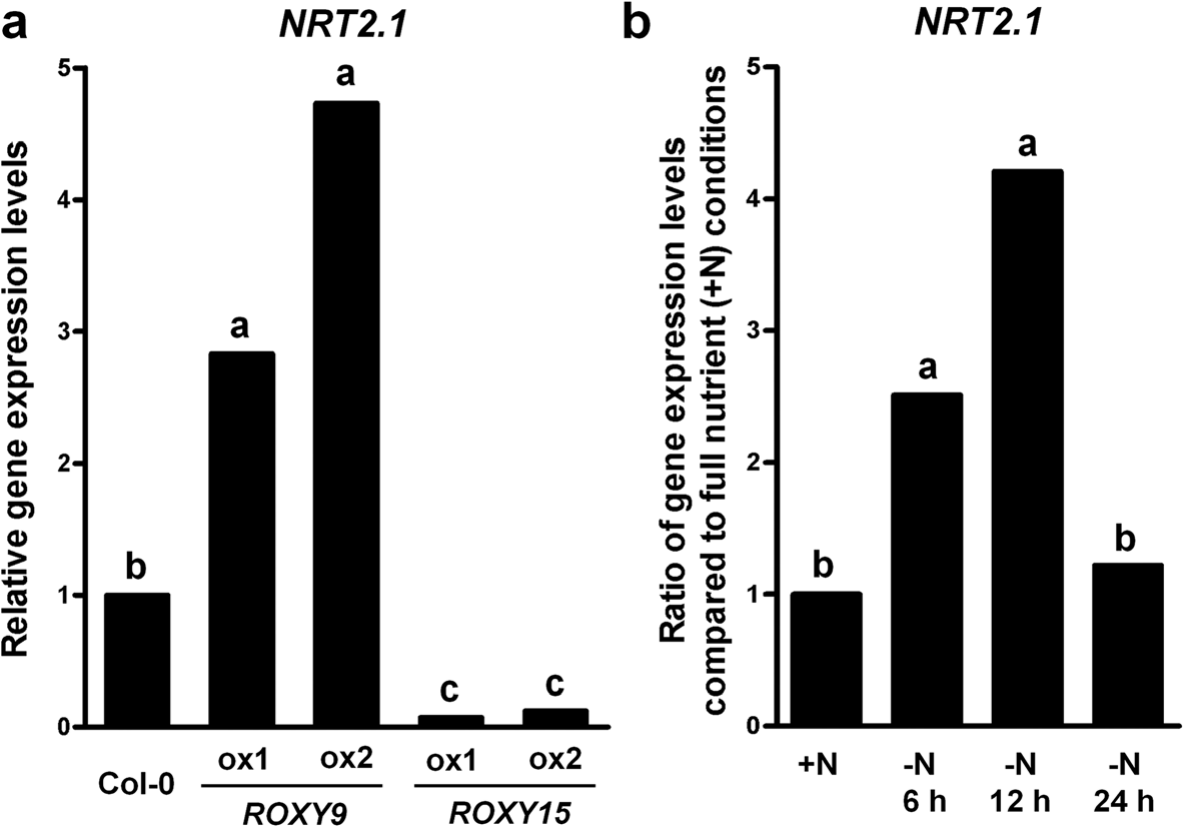
Overexpression of *ROXY9* causes the upregulation of the expression of *NRT2.1* while overexpression of *ROXY15* leads to the downregulation of the expression of *NRT2.1*. **a** Expression levels of *NRT2.1* in two independent transgenic seedlings overexpressing *ROXY9* or *ROXY15* (ox) relative to those in the wild type Col-0 seedlings (set to a value of 1). Seven-day-old seedlings grown on complete nutrient medium were used and the expression data were obtained by qRT-PCR. An *ACTIN7* was used as a reference gene. Different letters above the bars indicate values that are statistically different (*n* = 3 biological replicates, *P* < 0.05; *t* test). **b** Expression levels of *NRT2.1* in the wild type Col-0 seedlings under nitrate-deficient conditions. Expression levels of *NRT2.1* in Col-0 seedlings under nitrate-deficient conditions relative to those in Col-0 seedlings under nitrate-sufficient conditions (set to a value of 1). Six-day-old seedlings were grown on complete nutrient media and transferred to media with either 4 mM nitrate (+N) or no nitrate (-N), and further grown for 6, 12, and 24 h. Then, all seedlings were harvested at the end of the light period (zeitgeber time 16). An ACTIN7 was used as a reference gene. Different letters above the bars indicate values that are statistically different (*n* = 3 biological replicates, *P* < 0.05; *t* test)

To determine whether ROXYs mediate the transcriptional regulation under nitrate starvation, qRT-PCR was used to measure the mRNA levels of nitrate-related genes that are involved in nitrogen uptake in the two independent lines overexpressing *ROXY9* or *ROXY15*. Compared with the wild type seedlings grown on full nutrient medium, the expression of *NRT2.1* was significantly higher in the two independent lines overexpressing *ROXY9* and lower in the two independent lines overexpressing *ROXY15* (Fig. 4a). The change in *NRT2.1* transcriptional levels in wild type plants under nitrate deficiency was also investigated. The expression of *NRT2.1* was upregulated when plants placed on medium that did not contain nitrate for 6 h and 12 h (Fig. 4b).

### Nitrate starvation induces ROS production at root hair differentiation zone and ROS treatment differentially regulates the expression of *ROXYs*

**Fig. 5:**
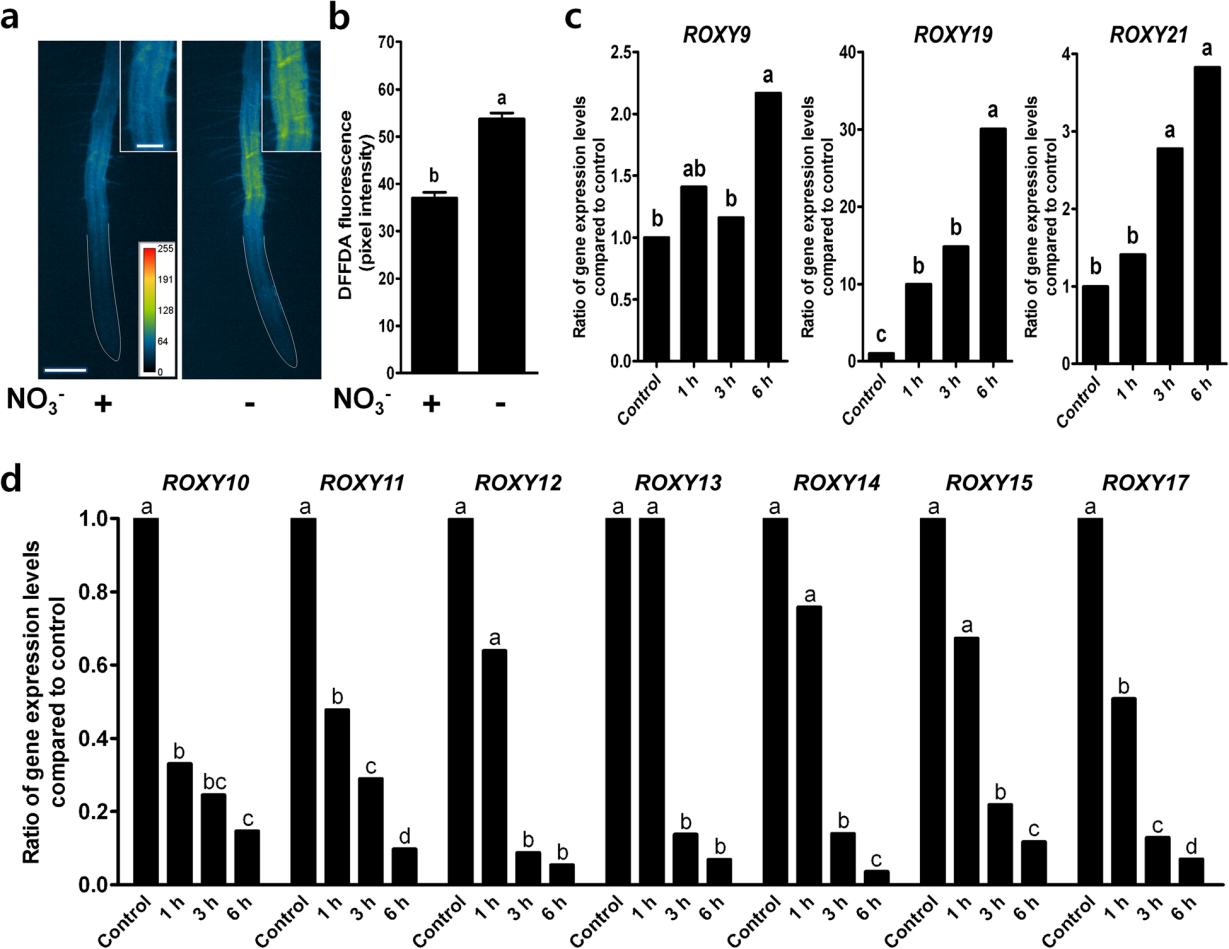
Nitrate deprivation induces ROS at the root hair differentiation zone (RHDZ) and H_2_O_2_ treatment alters the gene expression of *ROXY*s. **a** Pseudo-color images of ROS fluorescence are shown for nitrate-sufficient (4 mM NO_3_^−^) wild type roots and for nitrate-starved (no NO_3_^−^) wild type roots. Three-day-old seedlings grown on complete nutrient medium were floated onto liquid nutrient media containing either 4 mM nitrate or no nitrate. After 24 h, ROS images were collected following staining of roots with 20 μM DFFDA for 20 min. Yellow and red colors indicate higher ROS production (see a bottom inset for pixel intensity). Top insets show magnification of the RHDZ. White lines were drawn by hand to outline the root. Bar = 200 μm; bar in inset = 100 μm. **b** Quantified data of ROS in the RHDZ from representative images shown in (a) (*n* = 10 images of individual seedlings ± SE). Results from one of two independent experiments are shown here. Different letters above the bars indicate values that are statistically different (*P* < 0.05; *t* test). **c** H_2_O_2_ treatment upregulates *ROXY9,19,21* while (**d**) H_2_O_2_ treatment downregulates *ROXY10–15,17*. Expression levels of *ROXY* genes in the wild type seedlings treated with H_2_O_2_ relative to those in the wild type seedlings not treated with H_2_O_2_ (set to 1) are shown. Seven-day-old seedlings grown on complete nutrient medium were floated onto the same complete liquid nutrient media containing 10 mM H_2_O_2_ for 1, 3, and 6 h. Gene expression levels were quantified using qRT-PCR with *ACTIN7* as a reference gene. The fold change of *ROXY* expression levels in seedlings treated with H_2_O_2_ was compared with the *ROXY* expression levels in seedlings that were not treated with H_2_O_2_. Different letters above the bars indicate values that are significantly different (*n* = 3 biological replicates, *P* < 0.05; *t* test)

Nitrate starvation has been shown to induce about a 2-fold increase of ROS in *Arabidopsis* roots [22] implicating ROS as a component of the response to low concentrations of nitrate. To confirm the previous findings, ROS was measured in wild type seedlings under nitrate-sufficient conditions and nitrate-deficient conditions by staining roots with 5-(and 6-) carboxy-2′,7′-difluorodihydrofluorescein diacetate (DFFDA) that detects ROS [24]. Under nitrate-sufficient conditions, small amounts of ROS were detected mainly in the root hair differentiation zone (RHDZ) of the roots (Fig. 5a, b). Under nitrate-deficient conditions, ROS significantly increased in the RHDZ of the roots, showing that the results are consistent with previous findings [22]. To determine whether or not ROS regulates the expression of *ROXY* genes, we treated wild type seedlings with H_2_O_2_ and investigated the expression pattern of *ROXYs* in response to ROS. ROS treatment significantly increased the expression of *ROXY9,19,21* (Fig. 5c), whose expression was also enhanced by nitrate deprivation (Fig. 1a). The ROS treatment significantly reduced the expression of *ROXY10* through *ROXY15* and *ROXY17* (Fig. 5d), whose expression was reduced by nitrate deprivation (Fig. 1b). These data suggest that ROS may act upstream of the expression of the *ROXY* genes in nitrate deprivation signaling.

## Discussion

Despite the importance of nitrogen for plant growth and agricultural productivity, signal transduction pathways in response to changes in external and internal nitrogen concentrations in plants have not been fully elucidated. In this study, we analyzed the gene expression patterns of 16 out of 21 *ROXY* genes under nitrate deprivation conditions and found that all 16 *ROXY* genes are differentially regulated under nitrate starvation. A gain-of-function approach suggested a role for the ROXYs in the regulation of chlorophyll content, root hair growth, and transcription of *NRT2.1* under nitrate starvation conditions. We also showed that ROS production increases under nitrate deficiency and that ROS treatment also differentially regulates the expression of the *ROXY* genes. This work suggests possible roles of ROXYs and ROS in nitrate starvation signaling.

We showed that nitrate deprivation induces the differential regulation of *ROXY* genes: upregulation of 6 *ROXYs* (*ROXY6,8,9,19–21*) and downregulation of 10 *ROXYs* (*ROXY7,10–18*) (Fig. 1). Similar transcription pattern of some *ROXY* genes was found in a transcriptional profiling data set where 100 μM of nitrate was used to deprive plants [71]. It was also shown that the expression of 7 *ROXY* genes (*ROXY4,11–13,15–17*) was induced by the addition of nitrate [57] highlighting the importance of this gene family in nitrate response. Our data showing downregulation of the 6 *ROXYs* (*ROXY11–13,15–17*) under nitrate deprivation differ from previous findings [57] in that we studied response to nitrate deficiency rather than induction by high nitrate. In nitrate response for which nitrate is added to plants grown on media that used ammonium as a sole nitrogen source, the nitrate transporter NRT1.1 (CHL1/NPF6.3) turned out to be an important player which acts as a nitrate sensor that regulates the response to nitrate addition [72, 73]. We have some evidence that the differential regulation of *ROXYs* under nitrate starvation is altered in the mutants of *NRT1.1* only in the presence of ammonium (data not shown). These data indicate that NRT1.1 may also play a role in nitrogen starvation signaling and that nitrogen starvation response and response to nitrate addition may share some signaling components such as ROXYs and NRT1.1 under certain circumstances (e.g. in the presence of ammonium).

It appears that *ROXYs* are involved not only in plant responses to nitrate deprivation but also in plant responses to other low nutrients such as potassium, phosphorus, sulfur, and iron. We found that *ROXY7* and *ROXY16* are upregulated under low phosphate conditions, while *ROXY18* is upregulated under low potassium conditions (Additional file 2). Genevestigator microarray database also showed that *ROXY10* is upregulated by iron deprivation and *ROXY4* and *ROXY12* are downregulated by sulfur deprivation (data not shown). These data indicate that ROXYs may act as downstream regulators in plant signaling pathways triggered in response to nutrient deficiencies.

To understand the possible roles of ROXYs under nitrate-sufficient and nitrate-deficient conditions, we analyzed the phenotypes of transgenic lines overexpressing *ROXY9*, which is normally upregulated, and *ROXY15*, which is normally downregulated, under nitrate starvation conditions. We showed that transgenic lines overexpressing *ROXY9* had lower chlorophyll content compared to wild type plants while transgenic lines overexpressing *ROXY15* had higher chlorophyll content and that nitrate deficiency led to the decrease of chlorophyll content (Fig. 2). Consistent with our finding, it is known that nitrogen deficiency represses chlorophyll synthesis-related genes and the chlorophyll content is also reduced during low nitrogen conditions [71, 74–76]. These findings suggest that ROXY9 or its paralogs might play a role in chlorophyll synthesis as a negative regulator under nitrate-deficient conditions while ROXY15 or its paralogs might play a role in chlorophyll synthesis as a positive regulator under nitrate-sufficient conditions. The overexpression of *OsGRX6*, a rice CC-type *GRX* that shows a high similarity to *AtROXY18*, increased chlorophyll content and nitrogen content [56] supporting our finding that ROXYs may be involved in chlorophyll synthesis, which may depend on external nitrate status in plants.

We showed that transgenic lines overexpressing *ROXY9* had longer root hairs compared to wild type plants, while transgenic lines overexpressing *ROXY15* had shorter root hairs (Fig. 3a and b). Previously it was reported that nitrogen deprivation stimulates root hair elongation in *Arabidopsis* roots [77, 78]. Also, we found that roots deprived of nitrate for 1 d had longer root hairs compared to roots under nitrate-sufficient conditions (Fig. 3c) confirming the previous report. Based on these data, it is possible that ROXY9 or its paralogs might stimulate root hair elongation under nitrate-deficient conditions, while ROXY15 or its paralogs might inhibit root hair elongation under nitrate-sufficient conditions. These results also support a role for ROXY9 under nitrate-deficient conditions and ROXY15 under nitrate-sufficient conditions.

We also showed that the expression of *NRT2.1,* a major contributor in nitrogen uptake under nitrogen limitation [12], is differentially regulated in transgenic *ROXY9*- or *ROXY15*- overexpressor lines. The expression of *NRT2.1* was significantly upregulated in transgenic lines overexpressing *ROXY9* under full nutrient conditions but downregulated in transgenic lines overexpressing *ROXY15* (Fig. 4a). And nitrate deficiency in plants caused the upregulation of *NRT2.1* (Fig. 4b). Consistent with our data, it was shown that expression of *NRT2.1* is downregulated under nitrogen-sufficient conditions and upregulated by nitrogen starvation [12, 79–82]. Based on these findings, it is possible to speculate that ROXY9 or its paralogs might stimulate *NRT2.1* expression under nitrate-deficient conditions while ROXY15 or its paralogs might repress *NRT2.1* expression under nitrate-sufficient conditions which is consistent with our gene expression analyses. Likewise, the gain-of-function studies of *ROXY* genes provided some evidence about the possible roles of the *ROXY* genes in the regulation of gene expression and physiological change of plants in response to nitrate deficiency. As well as the gain-of-function studies, we also tried to use a loss-of-function approach by creating plants carrying RNAi constructs that specifically target one or multiple *ROXY* gene(s). Although we succeeded in making transgenic lines where a *ROXY9* was silenced or both *ROXY8* and *ROXY9* were silenced, we did not find any phenotype in these RNAi lines (data not shown) probably due to a high redundancy of the *ROXY* gene family in the *Arabidopsis* genome. Recently it was shown that silencing *ROXY15* and its homologs, which are nitrate-inducible, caused a long primary root phenotype in nitrate-sufficient conditions, indicating that ROXY15 and its homologs are involved in nitrate-mediated primary root growth [57]. Taken together, our work and previous studies provide new important clues as to the possible roles of ROXYs in plant signaling pathways under deprived or high nitrate conditions.

We showed in this work that nitrate starvation increases ROS production using DFFDA, which confirms our previous work [22]. Our additional new experiments show that H_2_O_2_ treatment downregulates *ROXY10–15* and *ROXY17* and upregulates *ROXY9,19,21* (Fig. 5). Moreover, we also found that *ROXY16* and *ROXY18*, which are induced by phosphate- and potassium deficiency, respectively, are also upregulated by ROS treatment while low phosphate-inducible *ROXY7* is not altered by ROS treatment (Additional file 2). These data indicate that ROS may act upstream of some ROXYs in nitrate starvation signaling as it has been shown for responses to low potassium [24]. However, we cannot exclude the possibility that there might be positive feedback regulation so that ROS-induced ROXYs may further stimulate ROS production. Respiratory Burst Oxidase Homolog C (RBOHC) NADPH oxidase RHD2 was shown to be responsible for the low potassium-induced ROS production and the expression of *HAK5*, a low potassium-inducible potassium transporter [22, 24]. These results with ROXY and our previous results [22] suggest that RHD2 is not sufficient alone for nitrate signaling since the differential regulation of ROXY genes in *rhd2* in response to nitrate deprivation was the same as that of ROXY genes in Col-0 wild type (Data not shown) and our previous results also showed that ROS production was attenuated, but not abolished in the *rhd2* mutant.

An open, related question is how ROXYs regulate their downstream targets. Our data using transgenic *ROXY* overexpressors reveal the complete opposite roles of ROXYs in plant phenotype and transcriptional regulation despite their high homology. GRXs are involved in disulphide bridge reduction or protein S-glutathionylation, a posttranslational modification, in plant cells [29, 30]. It was suggested that GRXs regulate many targets that are involved in various cellular processes including oxidative stress responses, nitrogen, sulfur, and carbon metabolisms, translation, and protein folding via the reduction of disulphide bridge [83]. GRXs-mediated protein glutathionylation may also be an important mechanism to regulate protein activities. In animals, GRXs were also shown to glutathionylate transcription factors leading to the alteration of their DNA binding activity [84]. In plants, CC-type GRXs ROXY1/2 and ROXY19 were reported to interact with TGA transcription factors and suggested to act as regulators of TGA transcription activity probably by glutathionylation that affects flower development and the SA/JA signaling, respectively [61, 62, 66]. A recent report suggested a possible role of ROXYs as adapter proteins for the assembly of transcriptional repressor complexes on TGA-regulated target promoters [68]. In this context it was shown that in response to nitrate TGA1 and TGA4 transcription factors play important roles in the regulation of gene expression of *NRT2.1* and *NRT2.2* and modulation of primary root length and lateral root density [85]. Based on our finding that the gene expression levels of *NRT2.1* were significantly altered in the *ROXY9* and *ROXY15* overexpression lines (Fig. 4), we hypothesize that the ROXYs that are differentially regulated under high and low nitrate conditions could regulate the gene expression of NRT2.1 probably either through ROXYs-mediated glutathionylation or disulphide bridge reduction of TGA1/4 or through acting as adaptor proteins, which may help recruit transcriptional complexes on TGA-regulated target promoters. Further studies will be necessary to test this hypothesis.

## Conclusion

**Fig. 6:**
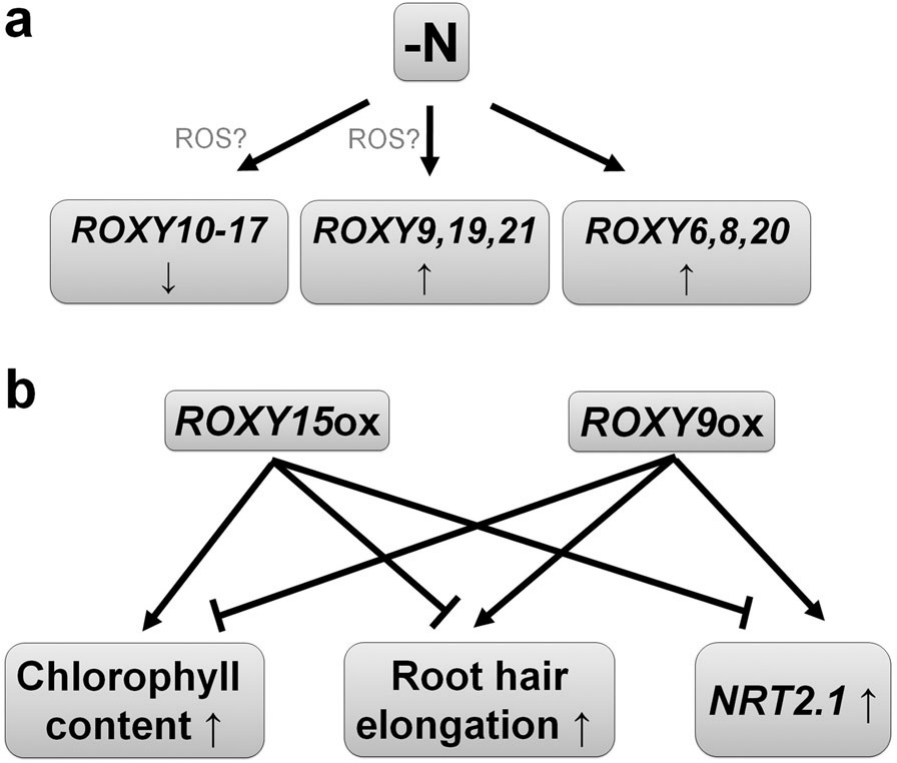
A model of the roles of ROXYs in nitrate starvation signaling. **a** Under nitrate-deficient conditions, *ROXY6,8,9,19–21* are upregulated, while *ROXY10–17* are downregulated. ROS may be a signaling component that regulates the expression of some of these *ROXY* genes. **b** Based on gain-of-function data, ROXY15 or its paralogs, which are upregulated under nitrate-sufficient conditions, might increase chlorophyll content and inhibit root hair elongation, and downregulate the transcription of *NRT2.1* while ROXY9 or its paralogs, which are upregulated under nitrate-deficient conditions, might reduce chlorophyll content and promote root hair elongation, and upregulate the transcription of *NRT2.1*. Up arrow, increase; down arrow, decrease; T-bar, inhibition

The data presented here provide evidence that both ROS and *ROXY* genes may play an important role in nitrate deprivation signaling. A model for the role of ROXYs in nitrate deprivation signal transduction pathway is summarized in Fig. 6. Our findings provide new and supportive data connecting known signaling components with nitrogen limitation-regulated plant responses (i.e. physiological changes and transcriptional changes) in nitrate deprivation signaling pathways. A major question still to be resolved is what the targets of ROXYs are and how ROXYs regulate their downstream targets in nitrate deprivation signal transduction pathway.

## Methods

### Plant materials and growth conditions

All *Arabidopsis thaliana* wild-type and transgenic lines were Columbia-0 ecotype. Seeds were sterilized in 70% (*v*/v) ethanol and 0.05% (v/v) Triton X-100 and then planted on 10 cm-diameter sterile plates containing complete nutrient medium [1.75 mM KCl, 2 mM Ca(NO_3_)_2_, 0.5 mM phosphoric acid, 0.75 mM MgSO_4_, 50 μM H_3_BO_3_, 10 μM MnCl, 2 μM ZnSO_4_, 1.5 μM CuSO_4_, 0.075 μM NH_4_Mo_7_O_24_, 74 μM Fe-EDTA, pH 5.6 with Ca(OH)_2_], 2% sucrose, and 0.8% SeaKem agarose (Cambrex). This complete nutrient medium was used throughout the study unless otherwise stated. After stratification of the seeds at 4 °C for 2~ 3 days, the plates were transferred to the growth chamber at 22 °C with a 16 h daylength at 200 μmol·m^− 2^ s^− 1^. Seedlings were grown on vertically oriented plates. Four- to 10-day-old seedlings were used throughout the study. For nitrate-deficient (-N) medium, 2 mM Ca(NO_3_)_2_ was replaced with 2 mM CaCl_2_.

### Plasmid construction and plant transformation

To generate constructs for the overexpression of *ROXY9* and *ROXY15*, full-length open reading frames of *ROXY9* and *ROXY15* were amplified by PCR using NEB Phusion polymerase. The PCR products were inserted into pENTR/D-TOPO following the manufacturer’s instructions (Invitrogen). The inserts were sequenced to make sure that no changes were introduced by PCR. The resulting entry clones were introduced into the destination plasmid pEARLYGATE100 [86] to yield *pEARLYGATE100:ROXY9* and *pEARLYGATE100:ROXY15*, which allows the overexpression of proteins under control of the cauliflower mosaic virus 35S promoter. Transgenic *Arabidopsis* plants carrying *pEARLYGATE100:ROXY9* and *pEARLYGATE100:ROXY15* were generated by Agrobacterium-mediated transformation [87] and T_3_ homozygous transgenic lines were used in this study.

### Quantitative real-time PCR analysis

For the analysis of *NRT2.1* expression levels in the wild type Col-0 seedlings and transgenic seedlings overexpressing *ROXY9* or *ROXY15*, seven-day-old seedlings grown on complete nutrient media were harvested. For the analysis of *NRT2.1* expression levels in Col-0 seedlings under nitrate starvation, six-day-old seedlings grown on complete nutrient media were transferred to media with either 4 mM nitrate or no nitrate and further grown for 6, 12, and 24 h. Then, all seedlings were harvested at the end of the light period (zeitgeber time 16). For the analysis of expression levels of *ROXY* genes in the wild type Col-0 seedlings with H_2_O_2_, seven-day-old seedlings grown on complete nutrient agar media were floated on the same nutrient liquid media containing 10 mM H_2_O_2_ for 1, 3, and 6 h and harvested at the same time. Total RNA was isolated by grinding whole seedlings in liquid nitrogen in the presence of Trizol reagent (Invitrogen) according to the manufacturer’s instructions. RNA was quantified and treated with RQ RNase-free DNase I (Promega). DNase-treated RNA was tested for genomic DNA contamination, and the quality of total RNA was determined by agarose gel electrophoresis. Two and a half micrograms of DNA-free RNA was then reverse transcribed using the First-Stand Synthesis System (Invitrogen). qRT-PCR analysis was performed using the StepOnePlus Real-Time PCR system (Applied Biosystems) and Platinum SYBR Green qPCR SuperMix-UDG (Invitrogen). The primers used to quantify the gene expression of *ROXYs* and *NRT2.1* were described in Additional file 3. The real-time PCR efficiency in the exponential phase was calculated according to the equation: E = 10^[− 1/slope]. Statistical differences of the transcript levels of *ROXY* genes and *NRT2.1* between samples were evaluated by a Student’s *t* test using ΔΔCt values [88]. Three biological replicates were used to generate means and statistical significance.

### ROS detection and measurement

For the localization and measurement of ROS in roots, we employed a membrane permeable fluorescent dye called 5-(and 6-) carboxy-2′,7′- difluorodihydrofluorescein diacetate (DFFDA, Invitrogen), an improved photostable version of 2′7’-dichlorodihydrofluorescein diacetate. For the ROS study using wild type Col-0 seedlings, three-day-old seedlings grown on complete nutrient media were floated on liquid media containing either 4 mM nitrate or no nitrate. After 23 h and 40 min, roots were further incubated with 20 μM DFFDA for 20 min. After a brief wash with the medium that did not contain DFFDA, the roots were observed using fluorescence microscopy. All fluorescence images were captured and stored in grayscale using a Nikon SMZ1500 microscope (Nikon,Tokyo, Japan) and a Q-Imaging Retiga cooled 12-bit camera (Burnaby, Canada) with 460–500 nm bandpass excitation and 510–560 nm bandpass emission. ROS fluorescence in the root hair elongation zone (~ 0.5 mm) was quantified and converted into pseudo-color images by the NIH ImageJ software program (available at rsb.info.nih.gov/ij/). Background noise was subtracted from the fluorescence intensity value for quantification. The same microscopic parameters (i.e., UV exposure time, gain, contrast, etc.) were used to compare ROS signal intensity within specific experiments.

### Chlorophyll content measurement

For the measurement of total chlorophyll content of *ROXY* transgenic plants, wild type Col-0 plants and two independent transgenic plants overexpressing *ROXY9* or *ROXY15* were grown in Fafard 4 M soil mix in a growth chamber at 22 °C with a 16 h daylength at 200 μmol·m^− 2^ s^− 1^ for 3 weeks. Photographs and chlorophyll content measurements of the plants mentioned above were taken. Chlorophyll contents were measured by bulking 2 fully expanded leaves at the same developmental stage from each of ten plants (*n* = 10). For the measurement of total chlorophyll content of plants deprived of nitrate, ten-day-old seedlings grown on complete nutrient media were transferred to media containing either 4 mM nitrate or no nitrate. The shoots were harvested 4 days after the transfer. Total chlorophyll content were measured by bulking 5 shoots per replicate and 5 replicates were used. Chlorophyll was extracted and assayed according to the procedure of Hiscox and Israelstam (1979). Statistical significance was evaluated with a Student’s *t* test. At least two independent experiments were performed, and similar results were obtained.

### Root hair length measurement

Root hair length was measured using Col-0 wild-type plants and two independent transgenic plants overexpressing *ROXY9* or *ROXY15*. Photographs of 4-day-old roots were taken using a Nikon SMZ1500 microscope and a Q-Imaging Retiga cooled 12-bit camera. For each root, the length of the root hairs, in a 2 mm region starting 0.5 mm above the root hair differentiation zone, was measured (*n* = 15 roots hairs), avoiding root hairs that were growing into the medium, by using NIH ImageJ software program. Ten plants per genotype were used for root hair length measurements (*n* = 10). For the root hair length measurement of plants deprived of nitrate, three-day-old seedlings were transferred to media containing either 4 mM nitrate or no nitrate. Then, root hair length was measured 24 h after the transfer. At least two independent experiments were performed, and similar results were obtained.

## Additional files

Additional file 1: **Figure S1.** Expression levels of *ROXY9* and *ROXY15* in transgenic overexpressor lines. (DOCX 118 kb)

Additional file 2: **Figure S2.** Expression levels of *ROXY18, ROXY7,* and *ROXY16* under low nutrients and H_2_O_2_ treatment. (DOCX 218 kb)

Additional file 3: **Table S1.** List of primers used in this research. (DOCX 28 kb)

## Abbreviations

DFFDA: 5-(and 6-) carboxy-2′,7′-difluorodihydrofluorescein diacetate
GRX: Glutaredoxin
NRT2.1: Nitrate transporter2.1
qRT-PCR: Quantitative reverse-transcription PCR
RHDZ: Root hair differentiation zone
ROS: Reactive oxygen species
ROXY: CC-type glutaredoxin
WT: Wild type
