# Supplementary material for: CC-type glutaredoxins mediate plant response and signaling under nitrate starvation in Arabidopsis
Source: BMC Plant Biol. 2018 Nov 13;18:281. doi: 10.1186/s12870-018-1512-1 (PMC6234535; doi:10.1186/s12870-018-1512-1)
Supplement: Supplementary file 1 — Figure S1. Expression levels of ROXY9 and ROXY15 in transgenic overexpressor lines. (DOCX 118 kb) [file 12870_2018_1512_MOESM1_ESM.docx]

**
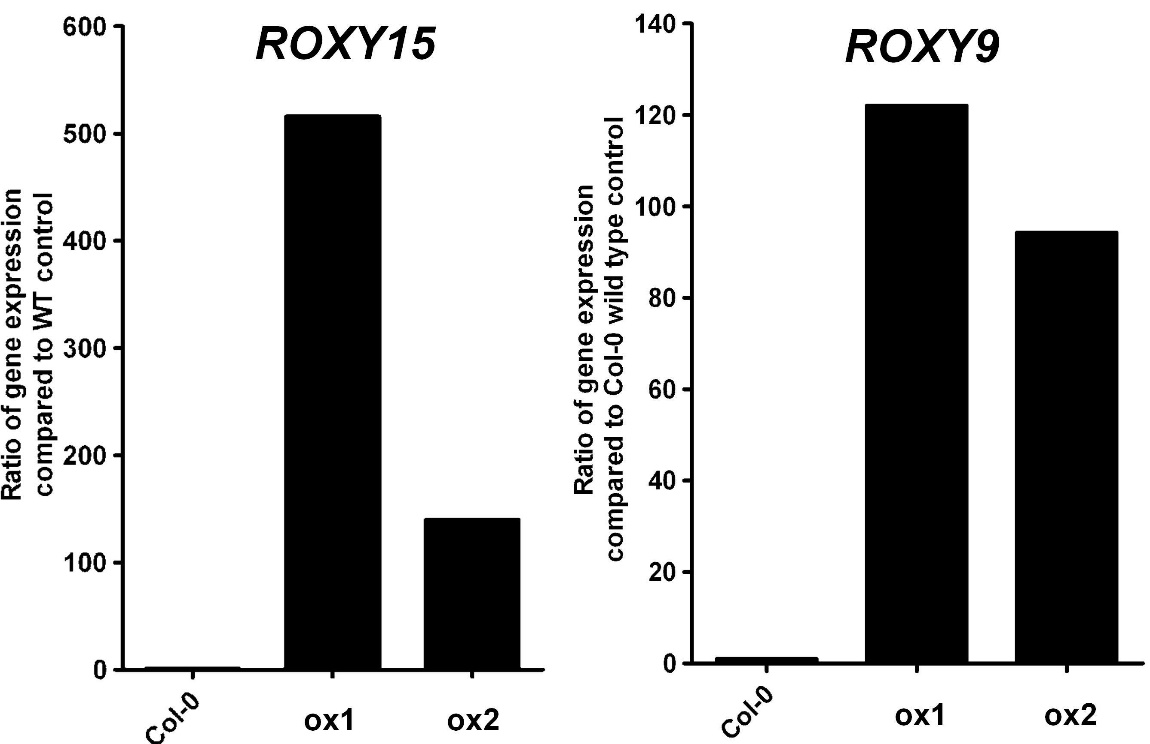
**

**Additional file 1: Fig. S1.** Expression levels of *ROXY9* and *ROXY15* in transgenic overexpressor lines.

Expression levels of *ROXY9* and *ROXY15* in two independent transgenic *ROXY9* or *ROXY15* overexpressor lines under nutrient-sufficient conditions relative to those in the wild type seedlings under nutrient-sufficient conditions (set to a value of 1). Seven-day-old seedlings were used and the expression data were obtained by qRT-PCR. An *ACTIN7* was used as a reference gene (n=1).
