# Supplementary material for: CC-type glutaredoxins mediate plant response and signaling under nitrate starvation in Arabidopsis
Source: BMC Plant Biol. 2018 Nov 13;18:281. doi: 10.1186/s12870-018-1512-1 (PMC6234535; doi:10.1186/s12870-018-1512-1)
Supplement: Supplementary file 2 — Figure S2. Expression levels of ROXY18, ROXY7, and ROXY16 under low nutrients and H2O2 treatment. (DOCX 218 kb) [file 12870_2018_1512_MOESM2_ESM.docx]

**
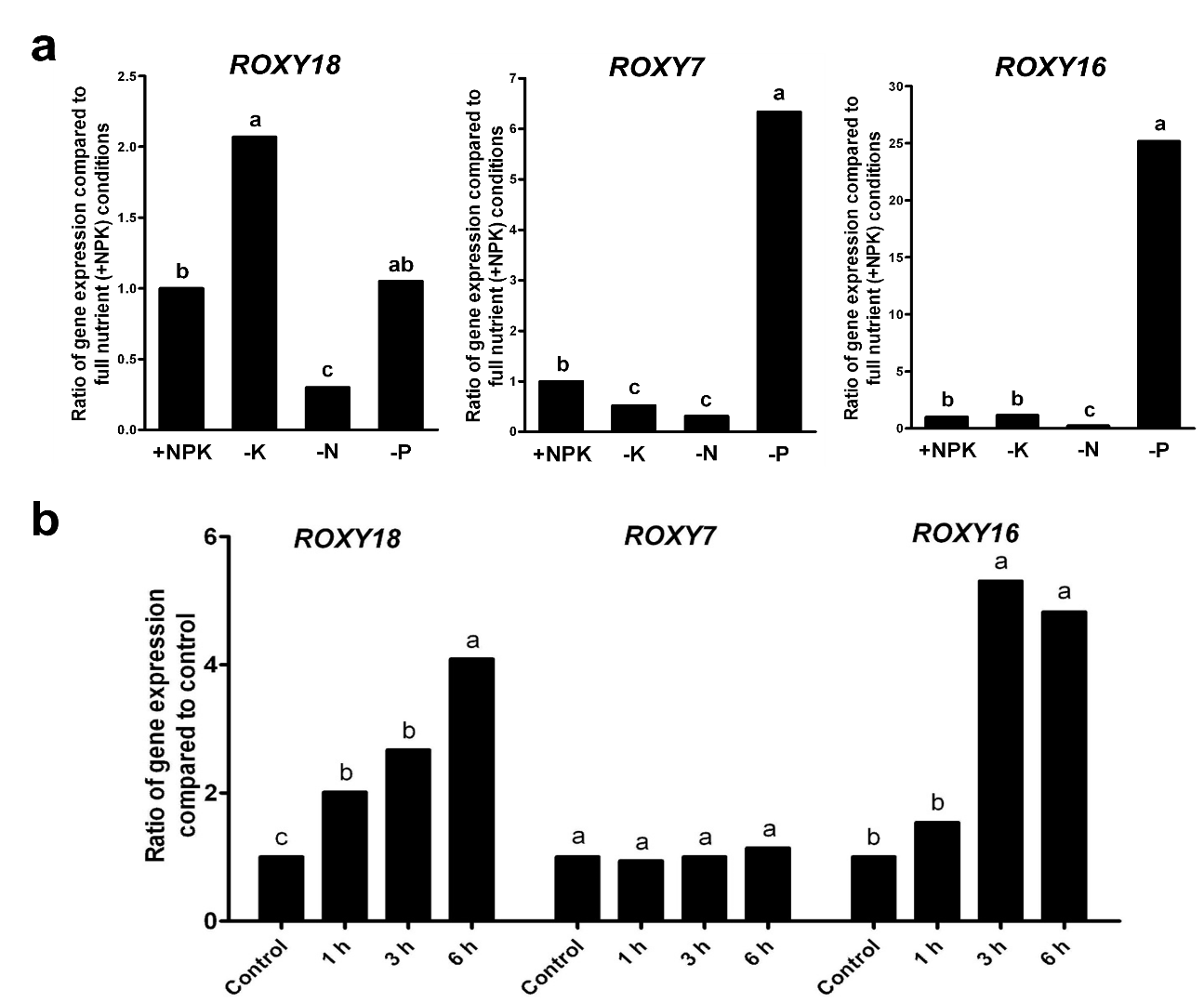
**

**Additional file 2: Fig. S2.** Expression levels of *ROXY18*, *ROXY7*, and *ROXY16* under low nutrients and H_2_O_2_ treatment.

(a) *ROXY18* is upregulated under potassium-deficient conditions, whereas *ROXY7,16* are upregulated under phosphate-deficient conditions. Seven-day-old seedlings grown under full nutrient conditions (+NPK) were deprived of either potassium (-K), nitrate (-N), or phosphate (-P) for 3 d. Expression levels of *ROXY* genes in the wild type Col-0 under low nutrients relative to those in the wild type seedlings under full nutrient conditions (set to the value of 1) are shown. The expression data were obtained by qRT-PCR. (b) Expression levels of *ROXY18*, *ROXY7*, and *ROXY16* in seedlings treated with H_2_O_2_ relative to those in seedlings not treated with H_2_O_2_ (set to a value of 1). Seven-day-old seedlings were treated with 10 mM H_2_O_2_ for 1, 3, and 6 h. The expression data were obtained by qRT-PCR. An *ACTIN7* was used as a reference gene. Different letters above the bars indicate values that are significantly different (n = 3 biological replicates, P < 0.05; *t* test).
