# Supplementary material for: CC-type glutaredoxins mediate plant response and signaling under nitrate starvation in Arabidopsis
Source: BMC Plant Biol. 2018 Nov 13;18:281. doi: 10.1186/s12870-018-1512-1 (PMC6234535; doi:10.1186/s12870-018-1512-1)
Supplement: Supplementary file 3 — Table S1. List of primers used in this research. (DOCX 28 kb) [file 12870_2018_1512_MOESM3_ESM.docx]

**Additional file 3: Table S1.** List of primers used in this research.

**Primers for the cloning of full-length *ROXY9* and *ROXY15***

|  | **Forward primer (5’ to 3’)** | **Reverse primer (5’ to 3’)** |
| --- | --- | --- |
| *ROXY9* (AT2G47880) | CACCATGGACAAAGTGATGAGAATGTCT | CTAGTAAAGGATGGACTGATAGGGTTTGATC |
| *ROXY15* (AT4G15660) | CACCATGGAGAAGATACAAAAGATGATCTCCGAG | TCAAAGCCATAAAGCCCCAAAGCGTTTAAG |

**Primers for qPCR**

|  | **Forward primer (5’ to 3’)** | **Reverse primer (5’ to 3’)** |
| --- | --- | --- |
| *ROXY6/AT1G06830* | CACCATGGACAAAGTTATGAGAATGTCGTCCGAA | TTAACATAAATATGGCTTCACTAGGGGAACG |
| *ROXY7/AT2G30540* | CACCATGGACAAAGTTGTGAGAATGTCGTCAGAG | TTAACATAGATTGGCTTGAAACGGCTTAAC |
| *ROXY8/AT3G62960* | CACCATGGACAAGGTTATGAGAATGTCATCGGAGA | CTAGTTATGAAATGACTGATACGGCTTGA |
| *ROXY9/AT2G47880* | CACCATGGACAAAGTGATGAGAATGTCT | CTAGTAAAGGATGGACTGATAGGGTTTGATC |
| *ROXY10/AT5G18600* | GCTGTTGGATCATATACCATATAAAATAGC | GAGCCGCAACAACGCCTGCTCGATCT |
| *ROXY11/AT4G15700* | GTCACTTCTCATTTTCACCCAAATATATCAA | GATAGATATTTGAAAACTGTCTTGCAAAGGAGA |
| *ROXY12/AT4G15690* | GTGAACAAAGAAAACATACACAAGAATGTATG | AGAACCAGTGTTTACAAAATAGGCAACCGGA |
| *ROXY13/AT4G15680* | ACTCTAAGTCATCTCTTATATATCGTCAGCCA | GGTATTATAATGTGAAATGTGTTCGAAGAGCGA |
| *ROXY14/AT4G15670* | CTATCTAAGTTTTGAAAGGATAGATTATGAGGCA | GGCATACACTTTTCTTAAATGCATGTTTGTATTC |
| *ROXY15/AT4G15660* | CAGTCTCCTTGATTGTCTCTAATTTTCCAATCT | CTTCTCCATTGGTGTTATAATGTAATAGGTT |
| *ROXY16/AT1G03020* | GATATTCAGCAAGACGTCCTGCTGTATG | TGCACCCAAGCTCTACAAGTGCTCGT |
| *ROXY17/AT3G62930* | GAGTTCCCACTCACATAGTCACACTAC | GAATTGGTCTAGCTCGTAGACCGTCAT |
| *ROXY18/AT1G03850* | CACCATGCAAAAAGCAATTCGACCATACGAGTC | TCAAAGCCATAAAGCCCCAGCTTGTCT |
| *ROXY19/AT1G28480* | CACCTGATTGTGATTGGACGGAGAGGATGTT | GGAACTAACTCACCGGAGATATGAGTA |
| *ROXY20/AT5G11930* | CACCATGAAGACGATGCGAGGTTTACGAAAC | TTATGCCCATAAGGCTCCAACCTCGACGAG |
| *ROXY21/AT4G33040* | ATGCGCGGCCGCTCATGAAGAGACTCTTAGCAACGAT | CCCGCGGCCGCTCATACCCAAAGAGCTCCAACTTGGA |
| *ACTIN7/AT5G09810* | ACCACTACCGCAGAAC | GCTCATACGGTCAGCA |
| *NRT2.1* | GGCTACGCATCTGACT | CGTGCAAGCGACTATC |
